# A hybrid imputation approach for microarray missing value estimation

**DOI:** 10.1186/1471-2164-16-S9-S1

**Published:** 2015-08-17

**Authors:** Huihui Li, Changbo Zhao, Fengfeng Shao, Guo-Zheng Li, Xiao Wang

**Affiliations:** 1 The Key Laboratory of Embedded System and Service Computing, Ministry of Education, Department of Control Science and Engineering, Tongji University, 201804 Shanghai, China; 2 Data Center of Traditional Chinese Medicine, China Academy of Chinese Medical Science, 100700 Beijing, China; 3 School of Computer and Communication Engineering, Zhengzhou University of Light Industry, 450002 Zhengzhou, China

**Keywords:** Microarray gene expression data, missing value imputation, large missing rate, Complement strategy, normalized root mean squared error

## Abstract

**Background:**

Missing data is an inevitable phenomenon in gene expression microarray experiments due to instrument failure or human error. It has a negative impact on performance of downstream analysis. Technically, most existing approaches suffer from this prevalent problem. Imputation is one of the frequently used methods for processing missing data. Actually many developments have been achieved in the research on estimating missing values. The challenging task is how to improve imputation accuracy for data with a large missing rate.

**Methods:**

In this paper, induced by the thought of collaborative training, we propose a novel hybrid imputation method, called Recursive Mutual Imputation (RMI). Specifically, RMI exploits global correlation information and local structure in the data, captured by two popular methods, Bayesian Principal Component Analysis (BPCA) and Local Least Squares (LLS), respectively. Mutual strategy is implemented by sharing the estimated data sequences at each recursive process. Meanwhile, we consider the imputation sequence based on the number of missing entries in the target gene. Furthermore, a weight based integrated method is utilized in the final assembling step.

**Results:**

We evaluate RMI with three state-of-art algorithms (BPCA, LLS, Iterated Local Least Squares imputation (ItrLLS)) on four publicly available microarray datasets. Experimental results clearly demonstrate that RMI significantly outperforms comparative methods in terms of Normalized Root Mean Square Error (NRMSE), especially for datasets with large missing rates and less complete genes.

**Conclusions:**

It is noted that our proposed hybrid imputation approach incorporates both global and local information of microarray genes, which achieves lower NRMSE values against to any single approach only. Besides, this study highlights the need for considering the imputing sequence of missing entries for imputation methods.

## Introduction

Gene expression profiling has been widely used in various studies over a wide range of biological disciplines, such as cancer classification, specific therapy identification, drug mechanism investigation [1]. However, data missing is an inevitable phenomenon in gene expression microarray experiment due to many factors, such as instrument failure, human error. Then, this situation will produce a negative impact on subsequent analysis. Many existing microarrays technologies, which require a complete data sequence as model input, have been disturbed to be put into practice for incomplete data [2], such as multivariate supervised classification methods (e.g. Support Vector Machines (SVMs) [3]), multivariate statistical analysis methods (e.g. Principal Component Analysis (PCA) [4] and Singular Value Decomposition (SVD) [5]).

In practice, there are three types of methods to process the genes with missing value before data analysis. The first method is to directly delete the missing genes, which may lead to information loss [6] as the missing genes may be diverse and some of them may play a crucial role in the subsequent analysis [7,8]. The second method is to replace the missing values by some simple numerical operations, such as imputed by zero, mean or mode of gene attributes [9]. Although this method requires quite a few computations, it may import error for the analysis of the studied mechanism. Actually, this approach would produce lots of same values, which is somewhat disagree with the situation of reality. The third method is to impute the missing values by their estimated values based on the observed information in the microarray dataset. The latest studies have shown that this method has strong adaptability and can obtain better imputation accuracy. Therefore, several methodologies have been developed in recent years [10].

Algorithms on imputing missing values can be classified into four categories [11,12]: global approach, local approach, hybrid approach and knowledge assisted approach. Each of them has its own characteristics. We will give a brief introduction on these approaches.

Global approach estimates missing values based on global correlation information extracted from the entire data matrix [12]. The frequently used global approaches include Singular Value Decomposition (SVD) [13] and Bayesian Principal Component Analysis (BPCA). These methods is characterized by the ability of capturing global correlation information to restore the missing values. But they ignore the local structure hidden in the data. Details of BPCA will be presented in later sections.

Correspondingly, local approach uses the potential local information to estimate the missing values, such as Local Least Squares (LLS) and Weighted K-Nearest Neighbor imputation (WKNN) [14]. LLS estimates missing values using a multiple regression model [11] based on K-nearest neighboring genes with respect to the target gene. Currently, several LLS variants have been proposed to improve algorithm performance in different aspects, such as Iterated Local Least Squares imputation (ItrLLS) [15], sequential Local Least Squares (sLLS) [16], weighted Local Least Squares (wLLS) [17]. Obviously, this type of method could estimate missing values accurately if the data matrix contains rich local structure. In other words, algorithms would obtain poor imputation performance when the missing values have strong correlation with the global information rather than local structure. In the next section, a short review of LLS and ItrLLS will be given.

Hybrid approach, apparently, is derived by combined both global and local correlations of the data matrix. Hence, using the hybrid method may achieve higher imputation performance than a single type approach only. LinCmb [18] and EMDI [19] are two typical hybrid approaches.

Knowledge assisted approach integrates domain knowledge or external information into the imputation process, which may significantly improve the imputation accuracy [12], such as Fuzzy C-Means clustering (FCM) [20] and Projection Onto Convex Sets (POCS) [21]. Wherein, FCM used gene ontology annotation as external information to process missing values imputation. However, the priori knowledge is difficult to extract and its veracity is hard to control. Thus, this problem causes poor applicability with these methods for data imputation.

Recently, many novel imputation algorithms have been developed, such as bicluster-based impute (BIC) [22], bicluster-based Least Square (bi-iLS) [23] and bicluster-based Bayesian Principal Component Analysis (bi-BPCA) [11]. Although they properly utilize data local structure and global correlation information inspired by bicluster algorithm, and a novel framework for missing value estimation is also designed, they still just use them separately without considering the advantage of their combination. Besides, most methods estimate missing values without considering the imputation sequence among incomplete genes. Since incomplete genes that have less missing entries might be recovered more accurately than those with more. The successful design of an estimation method for missing values depends mainly on making full use of the observed information. Thus the question is, how to build models using the different information and, more importantly, how the different methods can strengthen each other. Therefore, it is important to develop a strategy which could use the observed information fully for restoring the missing expression gene values.

In this paper, induced the thought of semi-supervised learning [24] with collaborative training, we propose a novel hybrid imputation method, called Recursive Mutual Imputation (RMI). In the field of machine learning and data mining, we always expect to collect a large amount of labeled data to build a powerful model. But obtaining large labeled data is time consuming and not practical for some fields, especially medical field. Yet, large unlabeled samples are often easy to obtain, making semi-supervised learning methods attractive. Moreover, semi-supervised learning can strengthen the model trained by labeled data through exploiting general knowledge among unlabeled samples. Co-training paradigm, one of a popular used semi-supervised method, is proposed by Blum and Mitchell [25]. It trains two classifiers with sufficient and redundant restrictions, respectively. But in reality, it is not easy to meet those two restrictions. Thus, in contrast to standard co-training configuration, CO-training REGressors (COREG) [26] has a broad applicability as no sufficient and redundant restrictions needed. COREG involves two classifiers, and the core idea of its training process is to select the confident labeled instances from dataset labeled by one learner, and then put them into another learner training set. Inspired by the idea of COREG, for a microarray dataset with a large missing rate, we treat the complete and incomplete genes as labeled and unlabeled examples correspondingly. Then, mutual imputation strategy is designed to share both global and local information extracted by two different types of imputation methods. To make full use of those information, an recursive imputation for data sequence is developed to improve all estimation results gradually. In our framework, Bayesian Principal Component Analysis (BPCA) and Local Least Squares (LLS) are introduced as our global and local approaches. Therefore, RMI is a hybrid imputation method, and has several distinguished advantages over other methods for restoring the missing expression gene values. Firstly, RMI aims to build a recursive mutual process by assembling two single methods, BPCA and LLS, which can exploits global correlation information and local structure in the missing dataset as full as possible. Secondly, it considers the genes that have less missing entries should be estimated firstly, which can improve the performance of estimation results for genes with more missing entries. Furthermore, a novel weight-based ensemble method is applied to RMI method. Experimental results conducted on several real-world datasets prove the effectiveness of the recursive mutual strategy, even in the case of larger missing rates and less complete genes.

The remainder of the paper is organized as follows. Section 2 reviews BPCA, LLS and ItrLLS approaches. Section 3 describes the proposed algorithm in detail. We presents the experimental results in Section 4. Finally, Section 5 concludes the work.

## Review on BPCA, LLS and ItrLLS

### Bayesian principal component analysis

BPCA [1] method is performed by three steps in the processes of missing value estimation, as follows: 1) Principal Component (PC) regression, 2) Bayesian estimation, and 3) an Expectation-Maximization (EM)-like repetitive algorithm. In detail, the following example is taken to explain how BPCA works. BPCA regards *d*-dimensional gene expression vectors *y *as a linear combination of principal axis

vectors *wl *(1 *≤ l ≤ K, and K < d*):

$$y=\overset{K}{\underset{l=1}{\sum }}x_{l}w_{l}+\varepsilon ,$$

where *x_l _*are called factor scores, *ε *denotes the residual error. The *l*th principal axis vector $w_{l}=\sqrt{\lambda_{l}}u_{l}$, where *λ_l _*and *µ_l _*denote the *l*th eigenvalue and the corresponding eigenvector of the covariance matrix *S *for the data set *Y *. The principal axis vectors *W *= (*W^obs^, W^miss^*), where *W^obs ^*denote the observed part in the data, and *W^mis ^*denote the miss part. If the number *K *is given, the factor scores *x *= (*x*_1_*, x*_2_*, . . . x_K _*) for the expression vector *y *can be obtained by minimizing the residual error over the observed data set *y^obs^*:

$$err=||y^{obs}-W^{obs}x|{|}^{2}.$$

Then, the missing value in the expression vector *y *can be estimated by:

$$y^{miss}=W^{miss}x.$$

The parameter *W *is unknown beforehand in the above procedure. BPCA use a probabilistic model, which is called probabilistic PCA (PPCA). Meanwhile, the model is based on the assumption that the residual error *ε *and the factor scores *x *obey normal distributions, as follows:

$$p\left(x\right)=N_{K}\left(x|0,I_{K}\right),$$

$$p\left(\varepsilon \right)=N_{d}\left(\varepsilon |0,\left(1/\tau \right)I_{d}\right),$$

where *I_K _*is a (K *× *K) identity matrix and *τ *is a scalar inverse variance of *ε*. N*K *(*x |u, σ *) denotes a *K*-dimensional normal distribution for *x*, whose mean and covariance are *u *and *σ*, respectively. BPCA assumes *Y *= {*Y^obs^, Y^mis^*}, where *Y^obs ^*and *Y^mis ^*denote observed part and the missing part. The variational Bayes algorithm [27] is used to estimate the posterior distribution of the parameter *θ ≡ *{*W, u, τ*} and *Y^mis ^*simultaneously. The value of *k *is *d − *1 as default.

### Local least squares

Two steps are included in the local least squares imputation procedure [2]: 1) selecting k genes based on Pearson correlation coefficients or Euclidean distance; 2) regression and estimation. Without loss of generality, let *Y *∈ *R*^*n×d *^denote the expression matrix consisting of *m *genes and *n *conditions, and the expression value of the *i*th gene in the *j*th condition is present as *gij *. Here we assume the target gene *gt *has missing values at the first *z *positions. As the following presents, there are *k *coherent genes (*g*_*t*1_, *g*_*t*2_, ... *g*_*tk*_) for *g*_*t *_in matrix *G*:

$$\left(\begin{array}{c} g_{t}\\ g_{t1}\\ \vdots \\ g_{tk} \end{array}\right)=\left(\begin{array}{cc} g_{1\times z} & w^{T}\\ B_{k\times z} & A_{k\times \left(n-z\right)} \end{array}\right),$$

where *B *denotes the *z *columns in the *k *coherent genes corresponding to the *z *missing positions of the target gene *g_t_*, and matrix *A *denotes the *n*-*z *columns in the *k *coherent genes corresponding to the *n*-*z *non-missing positions. *w^T ^*consists of the non-missing positions of *g_t_*. The *k*-dimensional coefficient vector *X *is regarded as a least squares problem with *A^T ^*and *w*, that is present as follow:

$$X=\text{arg}\underset{X}{\text{min}}|A^{T}X-w{|}^{2},$$

Then the *z *missing values in the target gene *g_t _*are estimated by a linear combination of *B^T ^*and *X *:

$$g_{1\times p}^{T}=B^{T}X=B^{T}{\left(A^{T}\right)}^{\nabla }w,$$

where (*A^T^*)^∇ ^denotes the pseudoinverse of *A^T^*. All the missing values in the matrix can be recovered according to the same procedure. Also LLS uses an artificial method to estimate the proper *k *mentioned above. In detail, for each target gene, the missing value is replaced by the row average at first. Secondly, a certain number of known entries are removed randomly to create the artificial missing matrix and save the true expression values. Then, LLS is performed on the artificial matrix using every value of *k *ranging from 1 to the total number of genes in the matrix. The imputation quality, measured by Normalized Root Mean Square Error (NRMSE) can be calculated once the pseudo missing values are estimated with every *k *value. Note that we know the true expression value for each of the pseudo missing value. Finally, the parameter *k *is set to the value corresponding to the best imputation quality.

### Iterated local least squares imputation

Iterated Local Least Squares imputation (ItrLLS) [15] method, one of LLS-derived methods, improves LLS method from two aspects. Firstly, the way of selecting coherent genes in ItrLLS is different from LLS. In LLS, the *k *coherent genes are selected as *k *nearest neighboring genes. The *k *value is fixed for all target genes after the *k *is selected at the artificial stage. While in ItrLLS, the number of coherent genes for target genes is not a fixed number. In practice, ItrLLS defines a distance threshold selecting the coherent gene, and the threshold *θ *is set as times the average distance to the target gene. Where *θ *denotes the distance ratio, which is chosen from a range [0.5, 1.5] with an increment 0.1 in the default setting. Secondly, ItrLLS uses iterative strategy. At each iteration, the estimated results from the last iteration is used to re-select coherent genes for every target gene with the same distance ratio, and then applies LLS method to re-estimate the missing values. The number of iterations is 5 as default.

## Recursive mutual imputation

To some extent, the single imputation method mentioned above works well in estimating missing values. They utilize local structure or global correlation information in the matrix properly. However, using a single type of usual imputation method may achieves less estimation accuracy than the hybrid method. Recently, several studies have shown that the performance of estimation algorithms is seriously constrained by the correlation information and structure in the data matrix [12,28].

In this paper, we propose a novel hybrid imputation method using recursive mutual strategy, called RMI. RMI aims to build a recursive mutual process by assembling two single methods, which could provide more accurate estimation. Two frequently- used methods, BPCA and LLS, are chosen as our baseline imputation algorithms. Since BPCA and LLS are able to capture global correlation information and local structure in the microarray matrix, respectively.

Below we describe the algorithm in detail. Let *C *and *M *denote complete genes set and incomplete genes set, extracted from the whole matrix *D *respectively. At the initial stage, the incomplete matrix *M *is divided into *p *parts, *M *= (*m*_1_*, m*_2_*, ... m_p_*)*^T ^*, where *m_i _*includes all genes with *i *missing entries, *p *is equal to the max number of missing entries. For example, for a matrix with 100 incomplete genes and 10 experiments, all genes that have one missing entries are selected as the gene group *m*_1_. Apparently, all genes that have two missing entries are selected as group *m*_2_, and the rest group can be formed in the same way. The *p *value might be less than 10 according to the attribute length of microarray data.

Generally, RMI consists of three major steps: two complete gene subsets construction; recursive mutual imputation and estimation results ensemble. Below we describe them in detail.

### Two complete gene subsets construction

The first step is to construct two complete genes subset, *C*1 and *C*2. As we discussed above, using the more observed information, the more accurate the estimation results. BPCA and LLS can be applied on the whole missing matrix *G *at the initial stage. Then, we obtain complete matrix *G_bpca _*and *G_lls_*. It is not proper to make *C*1 or *C*2 using all genes in *G_bpca _*and *G_lls_*, since they contain the genes with so many estimated value, which can induce more error. If *C*1 and *C*2 are both set to *C*, there would less observed information be used, which conflicts with our original view. Here, we set *C*1 by the part of genes in *G_bpca _*that have less than p*/*2 missing entries in the original matrix *G*. Similarly, *C*2 includes *C *and part of genes in *G_lls _*corresponding to the genes that have less than p*/*2 missing entries in *G*.

### Recursive mutual strategy

In the recursive mutual process, each subset can use information from each other for *C*1 and *C*2. We consider that incomplete genes should be recovered in the sequence of the size of *i*, which means RMI uses tactics to recover genes that have less missing entries firstly. In the process of semi-supervised co-training, a key step is to select the confident labeled instances from dataset labeled by one learner, and then put them into another learner training set. They can take respective advantages to improve the estimation results of each other in this way. In every iteration of RMI, the complete genes $m_{i}^{bpca}$ taken out from *C_bpca _*are regarded as confident genes, then we put them into complete genes set *C*2 which would be used in the next iteration of LLS imputation, where *C_bpca _*and *C_lls _*denote the imputed results by BPCA and LLS respectively. Correspondingly, $m_{i}^{lls}$ taken out from *C_lls _*should be added into complete genes set *C*1 in the next iteration of BPCA imputation. Following that, data imputation would stop until all missing entries in *M *are recovered. Note that RMI uses the complete genes' results from the last iteration.

At the end of RMI, the final complete matrix *G_im _*should be constructed by *C*1 and *C*2. We assemble the results using different weights for *C*1 and *C*2. The question is how to determine their weight. In the following, we define a novel confidence measure that suits our imputation model. Confidence in the imputation can be defined as the acceptance of imputation model to all estimation results. We consider that recursive mutual imputation tends to improve their accuracy as the amount of data over the observed entries grows. Actually, how well the estimator works directly depends on the number of observed entries in the missing gene and the correlation information between conditions. Based on this idea, we propose a simple definition for the confidence of each missing entry. For example, the target gene *g_t _*contains missing entries $g_{t}^{mis}$ and observing entries $g_{t}^{obs}\cdot g_{t}^{i}$ denotes one missing entry in $g_{t}^{mis}$, at the *i*th position. The confidence of $g_{t}^{i}$ in C1 can be calculated by the following equation:

$$CON\left(g_{t}^{i}\right)=\frac{1}{d_{i}*reg-error\left(g_{g}^{t}\right)},$$

where *d_i _*denotes the mean distance between $g_{t}^{obs}$ and its k nearest genes in *C*1. $reg_{e}rror\left(g_{t}^{i}\right)$ denotes the linear regression error, in which the *i*th position is the variable and the other positions in are variables. Note that these calculations are all based on the complete genes *C*1 or *C*2.

### Assembling the results

The confidence matrix *CON *(*M_bpca_*) and *CON *(*M_lls_*), corresponding to *C*1 and *C*2, can be obtained, according to the way we described above. The final step is to assemble the estimation results *C*1 and *C*2. Here the confidences can be used as the assembling weights, as following:

$$w_{bpca}^{ij}=\frac{CON{\left(M_{bpca}\right)}_{ij}}{CON{\left(M_{bpca}\right)}_{ij}+CON{\left(M_{lls}\right)}_{ij}},$$

$$w_{lls}^{ij}=1-w_{bpca}^{ij},$$

$$G_{im}^{ij}=w_{bpca}^{ij}*C1_{ij}+w_{lls}^{ij}*C2_{ij},$$

where *i *and *j *denote the missing positions in *G*. Algorithm 1 gives the algorithm framework.

**Algorithm 1 **RMI

Input:

The missing matrix *Gm×n *includes the complete genes set *C *and the incomplete genes set *M*.

Here *M *= (*m*_1_*, m*_2_*, ... m_p_*)*^T^*.

Output:

The complete matrix *G_im_*

Step 1: Constructing two complete genes subsets;

1: Two complete matrix *G_bpca _*and *G_lls _*can be obtained by using BPCA and LLS methods, respectively. The complete subset *C*1 is construct from *G_bpca_*. For each gene in *C*1, it has more than 1 and less than *p/*2 missing entries in the original matrix *G*. Similarly, The complete subset *C*2 is construct from *G_lls_*.

Step 2: Recursive mutual strategy;

2: **for ***i ∈ *1, 2*, ..., p ***do**

3: For each missing entries in the target gene *g_t _*in *m_i_*, obtain the confidences *CON_bpca_*(*g_t_*) and *CON_lls_*(*g_t_*) by *Eq*:

$$CON\left(g_{t}\right)=\frac{1}{d.*reg-error\left(g_{t}\right)},$$

4: Note that *CON_bpca_*(*g_t_*) is calculated based on *C*1 ∪ *C*, and *CON_lls_*(*g_t_*) based on *C*2 ∪ *C*.

5: **end for**

6: Recover the missing entries in *m_i _*based on *C*1 ∪ *C *using BPCA method, denoted $m_{i}^{bpca}$

7: Recover the missing entries in *mi *based on *C*2 ∪ *C *using LLS method, denoted $m_{i}^{lls}$

8: Update: $C1=C1\cup m_{i}^{lls}$; $C2=C2\cup m_{i}^{bpca}$;

Step 3: Assembling the results;

9: *G_im _*= *Assemble*(*C*1*, C*2) ∪ *C*

### Evaluation

#### Datasets

We used two types of microarray datasets, time series data and non-time series data to illustrate and evaluate RMI, following some previous studies [1,2,15]. Time series (TS) data includes three datasets, and two of them are used for identification of cell cycle-regulated genes of the yeast *Saccharomyces cerevisiae* (http://genome-www.stanford.edu/cellcycle/) [29]. The tab delimited data file contains three parts: the Alpha-part, cdc-part, and Elu-part. We choose Alpha-part and Elu-part. The first dataset, Alpha, contains 6075 genes in the original file. By removing genes with missing values, it remains 4489 genes and 18 experiments in total. The second dataset contains 5766 complete genes with 14 experiments, named Elu. The third dataset, Ronen, includes two time series in yeast from a study of response to environmental changes (http://ncbi.nlm.nih.gov/Projects/geo/query/acc.cgi?acc=GSE4158) [30], and is also used in [11] to assess bi-BPCA. The matrix contains 10749 genes in 26 experiments originally. It contains 5342 genes with 26 experiments finally, using the same preprocessing method mentioned in study [11]. The forth dataset, Tacrc, is non-time series dataset and is the cDNA microarray data relevant to human colorectal cancer (CRC) studied in [31]. It contains 758 genes with 50 experiments. The details of these four datasets are shown in Table 1.

**Table 1:** Testing datasets

	Original size	Complete size	Type
Alpha	6075*∗*18	4489*∗*18	Time Series
Elu	6075*∗*14	5766*∗*14	Time Series
Ronen	10749*∗*26	5342*∗*26	Time Series
Tacrc	758*∗*50	758*∗*50	Non-Time Series

#### Evaluation measures

#### The quality of the imputation results is measured by the Normalized Root Mean Square Error (NRMSE), which is described as following

$$NRMSE=\sqrt{\overset{N}{\underset{j=1}{\sum }}{\left(y_{j}-{\hat{y}}_{j}\right)}^{2}/N/\sigma_{y},}$$

where *y *is the real value and $\hat{y}$ is the value estimated by imputation method, and *σ_y _*is the standard deviation for the real values. *N *is equal to the total number of the missing entries. The smaller NRMSE is, the higher estimation accuracy.

#### Experimental setup

Three imputation methods, BPCA (http://www.csbio.sjtu.edu.cn/bioinf/EMDI/), ItrLLS (http://webdocs.cs.ualberta.ca/ghlin/src/WebTools/Imputation/) and LL-S (http://www.cs.umn.edu/hskim/tools.html) are selected as comparative methods with RMI. In our experiments, all the missing entries are generated based on the general principle. In a first step, the observations with missing values are deleted from the initial gene expression datasets to obtain testing datasets. We consider all complete genes and eliminate genes that have missing entries from the original datasets. Then artificial missing matrices with different missing rate are generated, and all the missing values in a dataset occur randomly. Let *t *denotes the complete ratio and *r *represents the missing rate. That means about *t*% of the whole genes in *G *are randomly selected as complete genes *C*, while *r*% of entries in the rest genes are removed randomly, denoted *M*. For example, assuming one matrix contains 1,000 genes, when *t *is equal to 0.20, then 200 genes are maintained as complete genes *C *and the rest 800 genes make up missing matrix *M *with *r*% of entries in *M *are removed. In a second step, different imputation methods are applied on these matrices. The evaluation performance between the estimated values and the original real values is calculated with criterion RMSE.

#### Results and analysis

**Figure 1:**
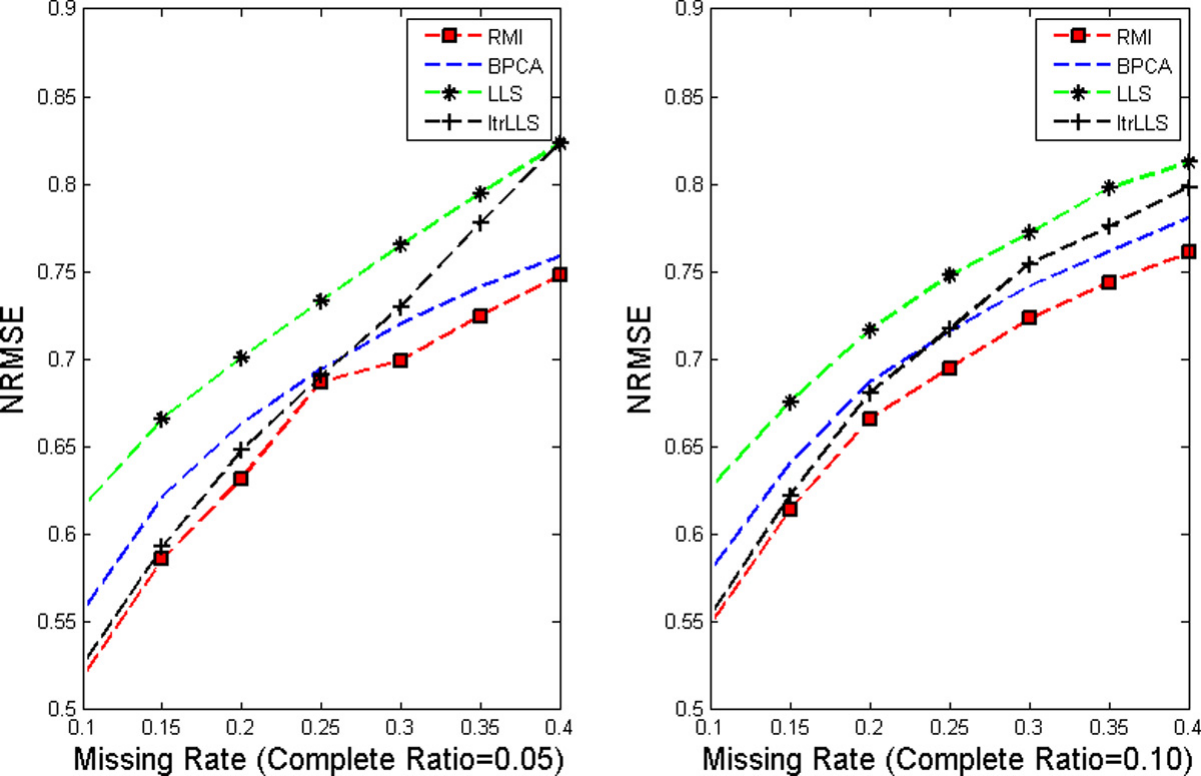
**NRMSE on Alpha**. The left and the right picture correspond to complete ratio at 0.05 and 0.25 respectively.

**Figure 2:**
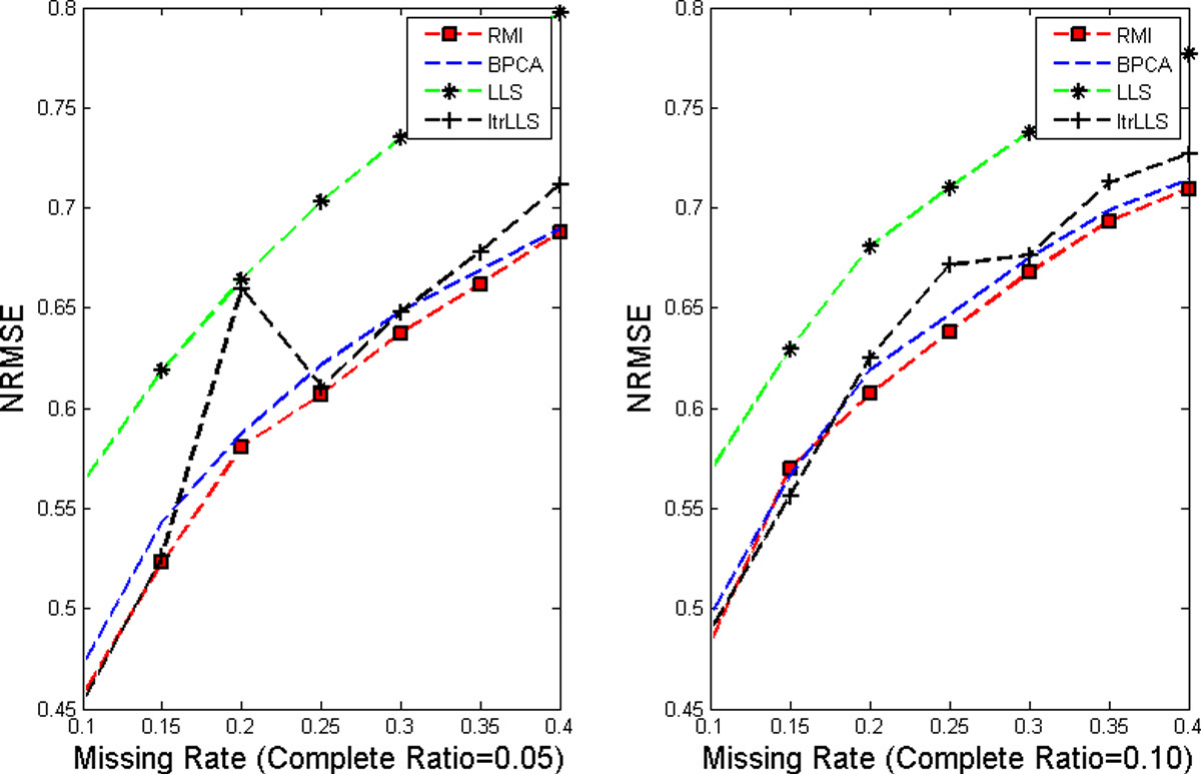
**NRMSE on Elu**. The left and the right picture correspond to complete ratio at 0.05 and 0.25 respectively.

**Figure 3:**
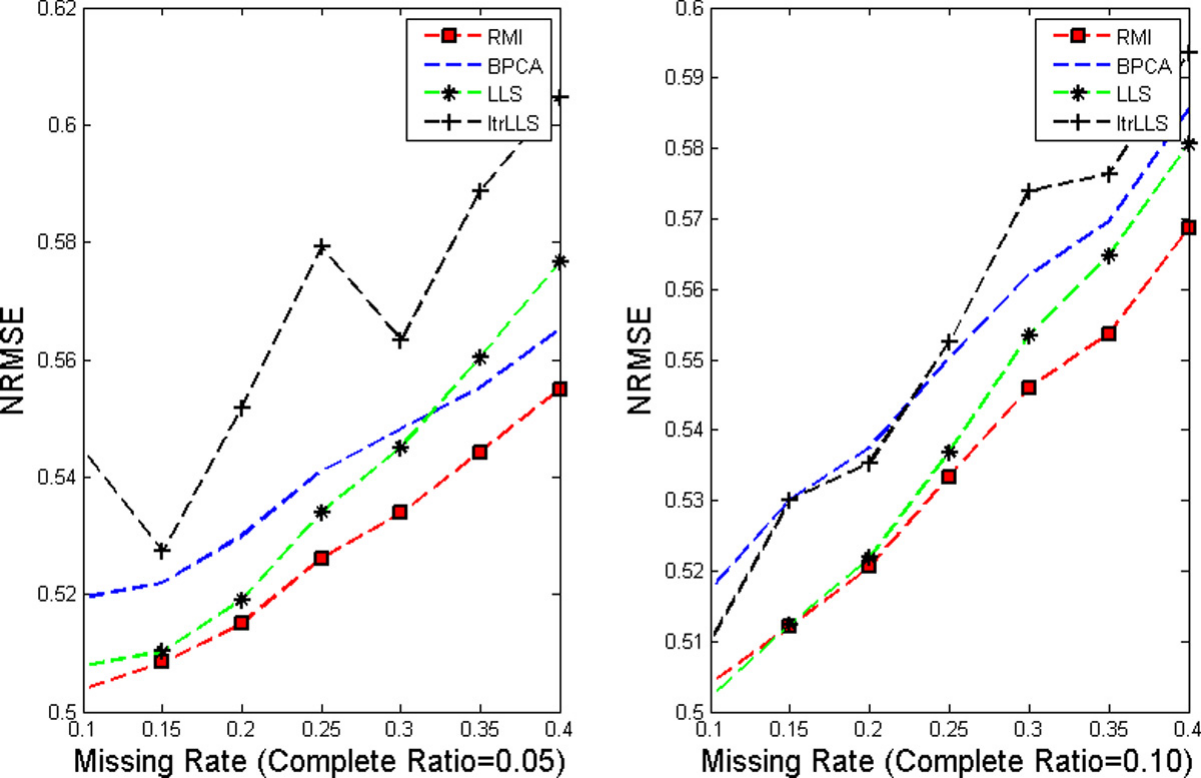
**NRMSE on Ronen**. The left and the right picture correspond to complete ratio at 0.05 and 0.25 respectively.

In this work, nine different missing rates *r *at 1%*−*40% with five different complete ratios *t *at 5%*−*25% are simulated in each dataset. Each kind of random case is generated 10 times by varying the set of missing entries to ensure a correct sampling. All the results present in the following figures are the NRMSE average value of 10 results. Figure 1, 2, 3, 4 show the results of four datasets: Alpha, Elu, Ronen and Tacrc. On the whole, it can be seen that RMI achieves the best performance by comparing these results for various missing rates *r*.

**Figure 4:**
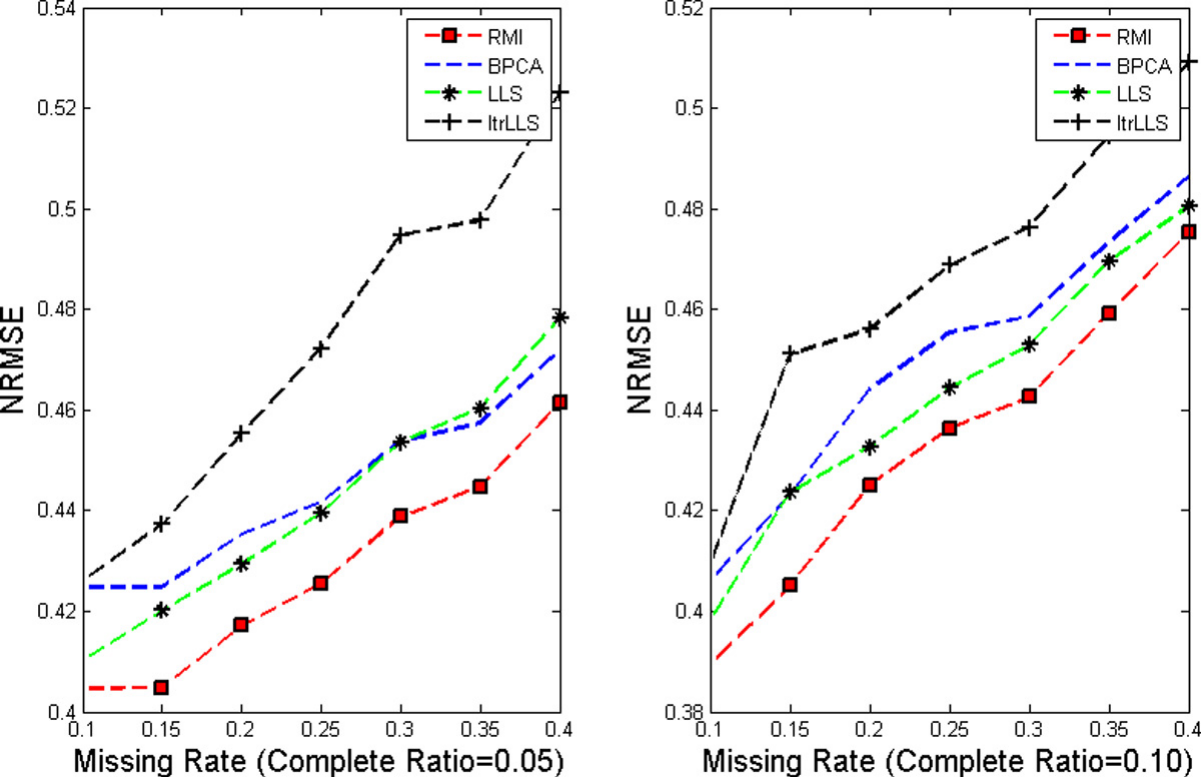
**NRMSE on Tacrc**. The left and the right picture correspond to complete ratio at 0.05 and 0.25 respectively.

**Figure 5:**
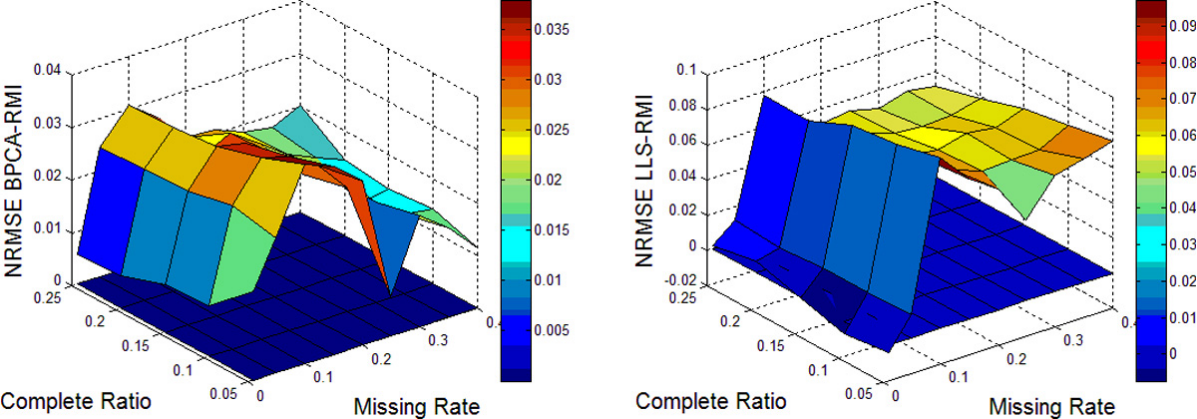
**NRMSE error on Alpha**. NRMSE error between the results of BPCA algorithm (left) or LLS algorithm(right) and RMI on the missing data problem for Alpha.

**Figure 6:**
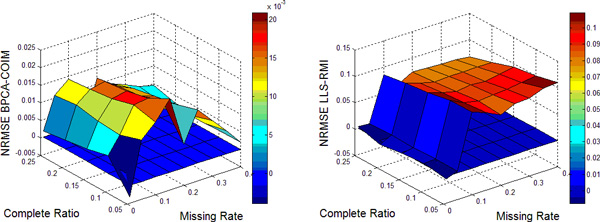
**NRMSE error on Elu**. NRMSE error between the results of BPCA algorithm (left) or LLS algorithm(right) and RMI on the missing data problem for Elu.

**Figure 7:**
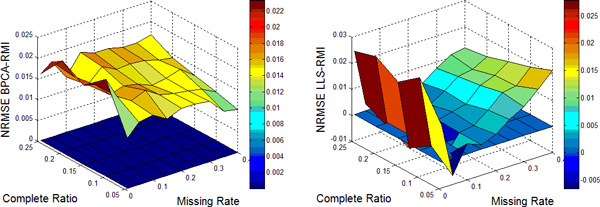
**NRMSE error on Ronen**. NRMSE error between the results of BPCA algorithm (left) or LLS algorithm(right) and RMI on the missing data problem for Ronen.

When comparing the three single imputation methods, there is no dominant method suitable for all datasets, different performances are present on the different datasets. Specifically, LLS is powerful for low rates of missing values on Ronen (seen from Figure 3). However, it should be noted that the efficiency of LLS is reduced when the missing rate increases. The same rule can be found from ItrLLS, Figure 3, 4 show us that ItrLLS obtains the lower NRMSE on env and Ronen, while it outperforms BPCA or ItrLLS on the other datasets, Alpha and Elu, especially in the low missing rates cases (seen from Figure 1 and Figure 2). Intuitively, RMI obtains the lower NRMSE on four datasets, which means it can maintain a stable performance, even for dataset with less complete genes and large missing rates. In order to see quantitative performance of RMI, Figure 5, 6, 7, 8 show the NRMSE error between the results of BPCA algorithm or LLS algorithm and RMI. Mathematically, NRMSE error is calculated by the BPCA NRMSE result or LLS NRMSE result minus the RMI NRMSE result on the same dataset. The greater is the *z *axis value, the better RMI performs. We can see that RMI method obtains lower N-RMSE than BPCA and LLS on the four datasets. Specifically, compared to BPCA, the NRMSE error averaged on all the test examples is improved by 2.16% on Alpha, 0.91% on Elu, 1.56% on Ronen and 1.71% on Tacrc, with RMI performing the best among the methods. For LLS, it is improved by 5.18% on Alpha, 6.79% on Elu, 0.77% on Ronen and 1.22% on Tacrc respectively. It can be seen that the worse results for the RMI occur occasionally. However, these just happens in the case of lower missing rates, besides it is hard to make a big gap between the performance of these imputation methods when few missing entries involved.

**Figure 8:**
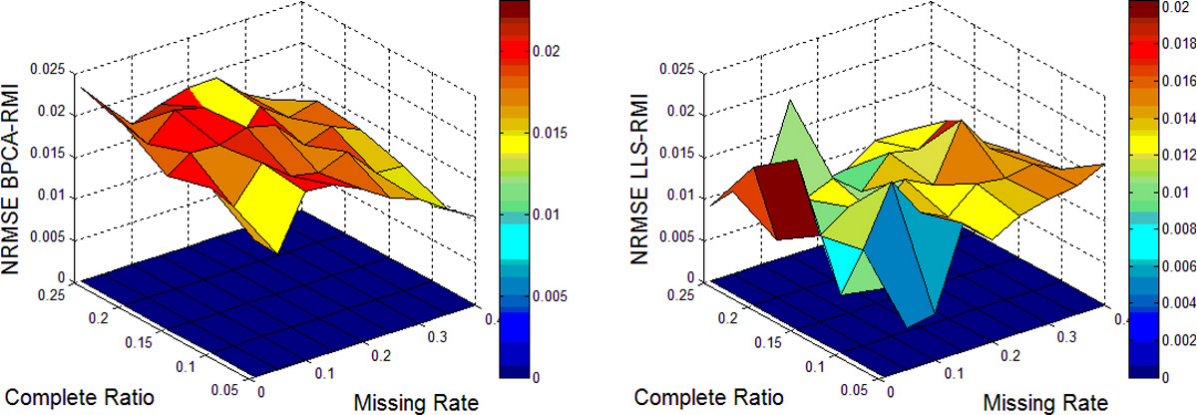
**NRMSE error on Tacrc**. NRMSE error between the results of BPCA algorithm (left) or LLS algorithm(right) and RMI on the missing data problem for Tacrc.

It should be noteworthy that RMI performs the lower NRMSE error than BPCA with the increase of missing rate *r *and decrease of complete ratio *t *(see the left figure of Figure 5, 6, 7, 8). On the contrary, it performs the higher NRMSE error than LLS with the increase of *r *and decrease of *t *(see the right figure of Figure 5, 6, 7, 8). This phenomenon is particularly prominent on Elu (see from Figure 6) and Ronen (seen from Figure 7). Because BPCA and LLS can make up for each other via the collaborative strategy in RMI. It also embodies the efficiency of the collaborative strategy. We consider that RMI outperforms comparative methods owing to using not only the local structures but also the global correlation information. Firstly, it aims to build a mutual process by assembling two single methods, BPCA and LLS, which can exploits global correlation information and local structure in the missing dataset as full as possible. Secondly, it considers the genes that have less missing entries should be estimated firstly, which can improve the performance of estimation for genes with more missing entries. Furthermore, a novel weight-based ensemble method is utilized in it.

## Conclusion

With the deepening research of DNA microarray, there produces a large number of microarray expression data. Missing values, as an important problem, has been influenced the research progress on this area. Numerous effective single methods have been proposed to estimate the missing values. However, they just uses the global information or local structure in the data matrix, which cannot fully used the observed information. In this study, inspired from collaborative training strategy, we propose a novel imputation method, called RMI. To our best knowledge, this work is the first attempt to focus on using an recursive mutual strategy to estimate missing values. Two frequently used methods BPCA and LLS are incorporated into RMI in order to fully exploit the observed information. In the process of the RMI, we consider the imputation sequence based on the number of missing entries in the target gene. Furthermore, a weight-based ensemble method is utilized in the final assembling step. We test four datasets to evaluate the performance of RMI. Various datasets with different missing rates are generated randomly for simulating the reality situation for modeling the real situation of missing matrix, randomly. Experimental results indicate that RMI is powerful and effective to impute missing values. And it is able to perform better performance than comparative methods even when the missing rate is large.

Some further research directions are worth for us to make a deeper study. It includes applying more sophisticated imputation methods in the recursive mutual strategy and to improve our current recursive mutual imputation framework. Another interesting issue is how to choose the right single methods in RMI. Meanwhile, the hybrid strategy is easily extended to develop a multiple hybrid version by using more than two single methods, which is a specific recommendation task.

## Competing interests

The authors declare that they have no competing interests.

## Authors' contributions

H Li and F Shao wrote the paper and done all the computational and analysis works. GZL conceived this paper and proposed the novel idea of RMI. C Zhao and × Wang participated in the design of the experiment. Also GZL and C Zhao revised the paper. All authors read and approved the final manuscript.

## Funding

This work and its publication were supported by the Natural Science Foundation of China under grant no. 61273305 and no. 81274007, no.61402422.

## References

[B1] Oba S Sato MA Takemasa I Monden M Matsubara K Ishii S A Bayesian missing value estimation method for gene expression profile data Bioinformatics 2003 19 16 2088 2096 10.1093/bioinformatics/btg287 14594714

[B2] Kim H Golub GH Park H Missing value estimation for dna microarray gene expression data: local least squares imputation Bioinformatics 2005 21 2 187 198 10.1093/bioinformatics/bth499 15333461

[B3] Hearst MA Dumais ST Osman E Platt J Scholkopf B Support vector machines IEEE 1998 13 4 18 28

[B4] Máckiewicz A Ratajczak W Principal components analysis (PCA) Computers & Geosciences 1993 19 3 303 342 10.1016/0098-3004(93)90090-R

[B5] Alter O Brown PO Botstein D Singular value decomposition for genome-wide expression data processing and modeling Proceedings of the National Academy of Sciences 2000 97 18 10101 10106 10.1073/pnas.97.18.10101 PMC27718 10963673

[B6] Statnikov A Aliferis CF Tsamardinos I Hardin D Levy S A comprehensive evaluation of multicategory classification methods for microarray gene expression cancer diagnosis Bioinformatics 2005 21 5 631 643 10.1093/bioinformatics/bti033 15374862

[B7] Celton M Malpertuy A Lelandais G De Brevern AG Comparative analysis of missing value imputation methods to improve clustering and interpretation of microarray experiments BMC Genomics 2010 11 1 15 10.1186/1471-2164-11-15 20056002 PMC2827407

[B8] Wang D Lv Y Guo Z Li X Li Y Zhu J Effects of replacing the unreliable cDNA microarray measurements on the disease classification based on gene expression profiles and functional modules Bioinformatics 2006 22 23 2883 2889 10.1093/bioinformatics/btl339 16809389

[B9] Allzadeh A Elsen M Davis R Chi M Lossos I Rosenwald A Distinct types of diffuse large B-cell lymphoma identified by gene expression profiling Nature 2000 403 6769 503 511 10.1038/35000501 10676951

[B10] Grużdž A Ihnatowicz A Ślkezak D Klopotek, M.A Gene expression clustering: Dealing with the missing values Intelligent Information Processing and Web Mining 2005 Springer, Gdansk, Poland 521 530

[B11] Meng F Cai C Yan H A bicluster-based bayesian principal component analysis method for microarray missing value estimation Biomedical and Health Informatics, IEEE Journal 2014 18 3 863 871 10.1109/JBHI.2013.2284795 24132028

[B12] Liew AWC Law NF Yan H Missing value imputation for gene expression data: computational techniques to recover missing data from available information Briefings in Bioinformatics 2011 12 5 498 513 10.1093/bib/bbq080 21156727

[B13] Yang YH Buckley MJ Dudoit S Speed TP Comparison of methods for image analysis on cDNA microarray data Journal of Computational and Graphical Statistics 2002 11 1 108 136 10.1198/106186002317375640

[B14] Troyanskaya O Cantor M Sherlock G Brown P Hastie T Tibshirani R Missing value estimation methods for DNA microarrays Bioinformatics 2001 17 6 520 525 10.1093/bioinformatics/17.6.520 11395428

[B15] Cai Z Heydari M Lin G Iterated local least squares microarray missing value imputation Journal of Bioinformatics and Computational Biology 2006 4 5 935 957 10.1142/S0219720006002302 17099935

[B16] Zhang X Song X Wang H Zhang H Sequential local least squares imputation estimating missing value of microarray data Computers in Biology and Medicine 2008 38 10 1112 1120 10.1016/j.compbiomed.2008.08.006 18828999

[B17] Ching WK Li L Tsing NK Tai CW Ng TW Wong A Cheng KW A weighted local least squares imputation method for missing value estimation in microarray gene expression data International Journal of Data Mining and Bioinformatics 2010 4 3 331 347 10.1504/IJDMB.2010.033524 20681483

[B18] Jörnsten R Wang HY Welsh WJ Ouyang M DNA microarray data imputation and significance analysis of differential expression Bioinformatics 2005 21 22 4155 4161 10.1093/bioinformatics/bti638 16118262

[B19] Pan XY Tian Y Huang Y Shen HB Towards better accuracy for missing value estimation of epistatic miniarray profiling data by a novel ensemble approach Genomics 2011 97 5 257 264 10.1016/j.ygeno.2011.03.001 21397683

[B20] Mohammadi A Saraee MH Estimating missing value in microarray data using fuzzy clustering and gene ontology Bioinformatics and Biomedicine, 2008. BIBM'08. IEEE International Conference 2008 IEEE 382 385

[B21] Gan X Liew AWC Yan H Microarray missing data imputation based on a set theoretic framework and biological knowledge Nucleic Acids Research 2006 34 5 1608 1619 10.1093/nar/gkl047 16549873 PMC1409680

[B22] Ji R Liu D Zhou Z A bicluster-based missing value imputation method for gene expression data Journal of Computational Information Systems 2011 7 13 4810 4818

[B23] Cheng KO Law NF Siu WC Iterative bicluster-based least square framework for estimation of missing values in microarray gene expression data Pattern Recognition 2012 45 4 1281 1289 10.1016/j.patcog.2011.10.012

[B24] Chapelle O Schölkopf B Zien A Semi-supervised learning 2006

[B25] Blum A Mitchell T Combining labeled and unlabeled data with co-training Proceedings of the Eleventh Annual Conference on Computational Learning Theory 1998 ACM 92 100

[B26] Zhou ZH Li M Semi-supervised regression with co-training-style algorithms Knowledge and Data Engineering, IEEE Transactions on 2005 19 11 1479 1493

[B27] Attias H Inferring parameters and structure of latent variable models by variational bayes Proceedings of the Fifteenth Conference on Uncertainty in Artificial Intelligence 1999 Morgan Kaufmann Publishers Inc 21 30

[B28] Brock GN Shaffer JR Blakesley RE Lotz MJ Tseng GC Which missing value imputation method to use in expression profiles: a comparative study and two selection schemes BMC Bioinformatics 2008 9 1 12 10.1186/1471-2105-9-12 18186917 PMC2253514

[B29] Spellman PT Sherlock G Zhang MQ Iyer VR Anders K Eisen MB Comprehensive identification of cell cycle-regulated genes of the yeast saccharomyces cerevisiae by microarray hybridization Molecular Biology of the Cell 1998 9 12 3273 3297 10.1091/mbc.9.12.3273 9843569 PMC25624

[B30] Ronen M Botstein D Transcriptional response of steady-state yeast cultures to transient perturbations in carbon source Proceedings of the National Academy of Sciences of the United States of America 2006 103 2 389 394 10.1073/pnas.0509978103 16381818 PMC1326188

[B31] Takemasa I Higuchi H Yamamoto H Sekimoto M Tomita N Nakamori S Construction of preferential cdna microarray specialized for human colorectal carcinoma: molecular sketch of colorectal cancer Biochemical and Biophysical Research Communications 2001 285 5 1244 1249 10.1006/bbrc.2001.5277 11478790

